# Associations of plasma sphingolipid profiles with insulin response during oral glucose testing in Icelandic horses

**DOI:** 10.1111/jvim.16200

**Published:** 2021-06-08

**Authors:** Yue Hei Leung, Ákos Kenéz, Anne Julia Grob, Karsten Feige, Tobias Warnken

**Affiliations:** ^1^ Department of Infectious Diseases and Public Health City University of Hong Kong Kowloon Hong Kong; ^2^ Clinic for Horses University of Veterinary Medicine Hannover, Foundation Hanover Germany

**Keywords:** ceramide, horse, insulin dysregulation, oral glucose test, sphingolipid, sphingomyelin

## Abstract

**Background:**

Sphingolipids modulate insulin sensitivity in mammals. Increased synthesis of ceramides is linked to decreased insulin sensitivity of tissues. Conversely, activation of the insulin signaling pathway can downregulate ceramide synthesis. Elucidating the association between sphingolipid metabolism and insulin response during oral glucose testing may help explain the pathophysiology of insulin dysregulation in horses.

**Hypotheses:**

Horses with insulin dysregulation will have a plasma sphingolipid profile characterized by increased ceramide concentrations. The plasma sphingolipid profile will have decreased ceramide concentrations after acute activation of the insulin signaling pathway by oral glucose testing.

**Animals:**

Twelve Icelandic horses.

**Methods:**

Horses were subjected to an oral glucose test (0.5 g/kg body weight glucose), with plasma insulin concentrations measured at 0, 30, 60, 120, 180, and 240 minutes postglucose administration. Plasma samples were collected at 0 and 120 minutes for sphingolipid profiling using a liquid chromatography‐mass spectrometry‐based metabolomics analysis. Eighty‐three species of sphingolipids were detected, including 3‐ketosphinganines, dihydroceramides, ceramides, dihydrosphingomyelins, sphingomyelins, galatosylceramides, glucosylceramides, lactosylceramides, and ceramide‐1‐phosphates.

**Results:**

Glucose administration did not significantly alter plasma sphingolipid profiles. C22:0‐ceramide, C24:1‐ceramide, C23:0‐ceramide, C16:1‐sphingomyelin, C22:0‐dihydroceramide, and C24:0‐ceramide were positively correlated with the insulin response (area under the curve).

**Conclusion and Clinical Importance:**

Positive correlation between the insulin response and sphingolipid concentrations implies upregulated sphingolipid metabolism in insulin dysregulated horses. A high plasma ceramide concentration can indicate insulin dysregulation in horses.

**Abbreviations**
Akt: protein kinase B
AUC_ins_
: area under the insulin response curve over time
C1P: ceramide‐1‐phosphate
Cer: ceramide
CerS: ceramide synthase
DHCer: dihydroceramide
DHSM: dihydrosphingomyelin
FDR: false discovery rate
GalCer: galatosylceramide
GluCer: glucosylceramide
ID: insulin dysregulation
Keto: 3‐ketosphinganine
LacCer: lactosylceramide
OGT: oral glucose test
PI3K: phosphatidylinositol 3‐kinase
SM: sphingomyelin

## 1 INTRODUCTION

Increasing attention has been directed to the pathophysiology of sphingolipid metabolism because of their cell signaling potential for modulating insulin sensitivity, apoptosis,^1^ and inflammation.^2^ Specifically, ceramides were identified as biomolecules inhibiting insulin action by dephosphorylating protein kinase B (Akt), a key element of the intracellular insulin signaling pathway.^3^, ^4^ Although the molecular actions of sphingolipids have not yet been confirmed in horses, a higher ceramide concentration is likely to decrease peripheral insulin sensitivity, leading to hyperinsulinemia instead of a normal insulin response. Thus, increased risk of insulin dysregulation (ID) is plausible in horses that have increased plasma ceramide concentrations, considering the action of ceramides on insulin signaling in mammalian cells.^3^ In horses, the most common clinically relevant sequela of ID is laminitis,^4^ which has been linked to ID and hyperinsulinemia in previous studies.^5^, ^6^, ^7^, ^8^ Furthermore, laminitis and laminar separation were shown to be associated with altered tissue sphingolipid concentration.^9^, ^10^ These findings suggest that sphingolipids, especially ceramides, could play a pathophysiological role in ID, impacting insulin sensitivity and metabolic health in horses.

**FIGURE 1:**
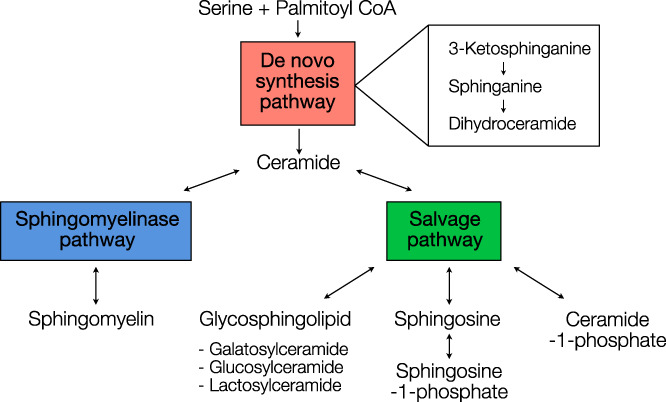
An overview of sphingolipid metabolic pathways, according to Merrill^11^ and Maceyka and Spiegel.^2^ The synthesis of ceramide begins with the condensation reaction of serine and palmitoyl‐CoA to form 3‐ketosphinganine. Along the de novo synthesis pathway (red), 3‐ketosphinganine is reduced to sphinganine. Sphinganine is coupled with fatty acyl‐CoA to form dihydroceramide, and a double bond is added on dihydroceramide to form ceramide. Ceramide is the central hub of the sphingolipid metabolism, which can be modified into sphingomyelin through the sphingomyelinase pathway (blue) and can be modified into glycosphingolipids or phosphorated sphingolipids through the salvage pathway (green). Double‐headed arrows indicate a reversible reaction

Bidirectional crosstalk occurs between the sphingolipid metabolic pathway (illustrated in Figure 1) and the insulin signaling pathway.^2^, ^11^ Constitutive activation of phosphatidylinositol 3‐kinase (PI3K), a key regulator of the insulin signaling pathway, can inhibit the sphingomyelinase (SM) pathway and suppress ceramide biosynthesis.^12^ Furthermore, the mechanistic target of rapamycin (mTOR) signaling pathway, which overlaps with the insulin signaling network, can inhibit de novo sphingolipid synthesis by orosomucoid protein phosphorylation.^13^ Conversely, ceramides can attenuate insulin signaling by dephosphorylation of Akt at Ser473,^14^ and ceramide‐1‐phosphate (C1P) can activate PI3K and Akt by rapid phosphorylation.^15^, ^16^ Thus, based on the crosstalk between sphingolipid metabolism and insulin signaling, glucose administration during oral glucose testing (OGT) may perturb sphingolipid metabolism and alter the plasma sphingolipid profile.

The OGT is commonly used to classify insulin response in horses.^17^, ^18^ The increased plasma glucose concentration during OGT might impair insulin response in ID if insulin resistance in peripheral tissues leads to excessive insulin secretion and hyperinsulinemia. If this insulin resistance is caused by increased ceramide concentrations, it could explain the link between alteration in sphingolipid metabolism and ID. However, the impact of an acute OGT challenge on sphingolipid metabolism in horses has been characterized only for a limited number of sphingolipids, mainly SM.^19^, ^20^, ^21^ The correlation between sphingolipids and insulin response has not yet been well established. We hypothesized that the OGT would alter plasma sphingolipid profiles, specifically that the acute activation of the insulin signaling pathway by an OGT would downregulate ceramide biosynthesis. Furthermore, we hypothesized that horses affected by ID during OGT would have a sphingolipid profile characterized by an increased ceramide concentration. We aimed to provide a comprehensive characterization of the plasma sphingolipid profile of healthy and hyperinsulinemic horses during OGT. Our objectives were to assess the short‐term impact of OGT on sphingolipid metabolism by comparing the plasma sphingolipid profiles at 0 and 120 minutes, relative to glucose administration, and to evaluate the correlation between sphingolipids and the insulin response during OGT.

## 2 MATERIALS AND METHODS

### 2.1 Horses

Twelve Icelandic horses (8 mares, 3 geldings, and 1 stallion) aged 15 to 29 years were enrolled in the study, including healthy (insulin sensitive) horses and horses previously found to be affected by ID based on PO dynamic testing. The horses had a median body weight (BW) of 400 kg (range, 230‐451 kg) and a median body condition score of 6 (range, 3‐8). The horses were kept in paddocks and were stabled in boxes overnight. They were fed hay ad libitum with no dietary supplements. The study was approved by the State Office for Consumer Protection and Food Safety in accordance with the German Animal Welfare Law (Ref: 33.19‐42502‐04‐18/3006).

### 2.2 Oral glucose test

The horses were fasted overnight for approximately 12 hours before testing. For collection of blood samples, a jugular vein catheter (Intraflon 12 G, Vygon, Ecouen, France) was aseptically inserted into a jugular vein 1 hour before sampling and glucose administration. The OGT was performed by nasogastric tube administration of 0.5 g/kg BW glucose (Glucose, WDT, Garbsen, Germany) dissolved in 2 L of water, as reported previously.^22^, ^23^ Blood samples were collected immediately before glucose administration, and at 30, 60, 120, 180, and 240 minutes after glucose administration.

### 2.3 Insulin quantification

Plasma insulin concentrations were measured in duplicate using an insulin ELISA designed for horses (Mercodia AB, Equine Insulin ELISA, Uppsala, Sweden) following the manufacturer's instructions. In cases of insulin concentrations exceeding the upper range of quantification, plasma samples were diluted 1:4 using the manufacturer's commercially available diabetes sample buffer (Mercodia, Diabetes Sample Buffer, Mercodia AB). User‐calculated intra‐assay coefficients of variation were 4.6%, and 1.9% in the low and high concentration ranges, respectively. Inter‐assay coefficients of variation were 9.7%, 6.9%, and 5.2% in the low, medium, and high concentration ranges, respectively.

### 2.4 Sphingolipid profiling

Equine plasma samples, collected at 0 and 120 minutes, relative to glucose administration, were used for sphingolipid analysis at The Metabolomic Innovation Centre (TMIC UVic Node at The University of Victoria, Genome BC Proteomics Centre, Victoria, British Colombia, Canada), as reported previously.^24^ Briefly, 50 μL of sample were mixed with 0.95 mL of methanol‐chloroform (5:2), including 0.1 mg/mL of butylated hydroxytoluene as antioxidant. The sample mixtures then were vortexed for 30 seconds, sonicated for 5 minutes in an ice bath, and centrifuged for 20 minutes at 21 000*g* at 10°C to precipitate proteins. The remaining pellet was extracted with 500 μL of chloroform (1:1, v/v) again, following the same procedure. The clear supernatants were pooled and dried with nitrogen gas at 30°C. The residues were redissolved in 1 mL of a methanol‐chloroform solution (1:1, v/v).

To identify and quantify the sphingolipids in the plasma samples, sphingolipid profiling was performed using ultraperformance liquid chromatography‐tandem mass spectrometry in multiple reaction monitoring mode, in which the sphingolipids were targeted in positive ion mode and the phosphorylated sphingolipids were targeted in negative ion mode. The concentration of detected sphingolipids was calculated using the measured peak area in the linear regression calibration curves.

### 2.5 Statistical analysis

To visualize the sphingolipid distribution before and after OGT, bar plots of 3 sphingolipid pathways were drawn in GraphPad Prism version 9.0.0 for Mac (GraphPad Software, La Jolla, California). The standard deviation was shown as the error bar. To visualize and quantify each horse's insulin response during OGT, the line plot and the area under the curve of each horse's insulin response (AUC_ins_) were calculated in GraphPad Prism 9. To evaluate the correlation between sphingolipids and insulin response, Pearson's correlation analyses were performed using MetaboAnalyst 4.0 (http://www.metaboanalyst.ca),^25^ with AUC_ins_ as the feature of interest. One sample *t*‐tests were performed to test that the correlation (*R*) was distinct from zero. The *P*‐values from the *t* test, comparing 0 and 120 minutes sphingolipid concentrations, were adjusted by false discovery rate (FDR) correction using the Benjamini‐Hochberg procedure.^26^ Individual correlation plots with simple linear regression were drawn using GraphPad Prism 9. The 95% confidence intervals of the best‐fit line were shown as dotted lines. The performance of the line of best fit was indicated by the coefficient of determination (*R* ^2^).

## 3 RESULTS

### 3.1 Acute impact of OGT on sphingolipid metabolism

In the sphingolipid profiling, 100 species of sphingolipids were targeted, and 86 species were detected, including 4 species of 3‐ketosphinganines (Keto), 5 species of dihydroceramides (DHCer), 11 species of ceramides (Cer), 13 species of dihydrosphingomyelins (DHSM), 19 species of SM, 8 species of galatosylceramides (GalCer), 4 species of glucosylceramides (GluCer), 12 species of lactosylceramides (LacCer), and 10 species of C1P. To assess the acute impact of OGT on sphingolipid metabolism, the concentration of each sphingolipid before and after OGT was plotted according to its pathway.

**FIGURE 2:**
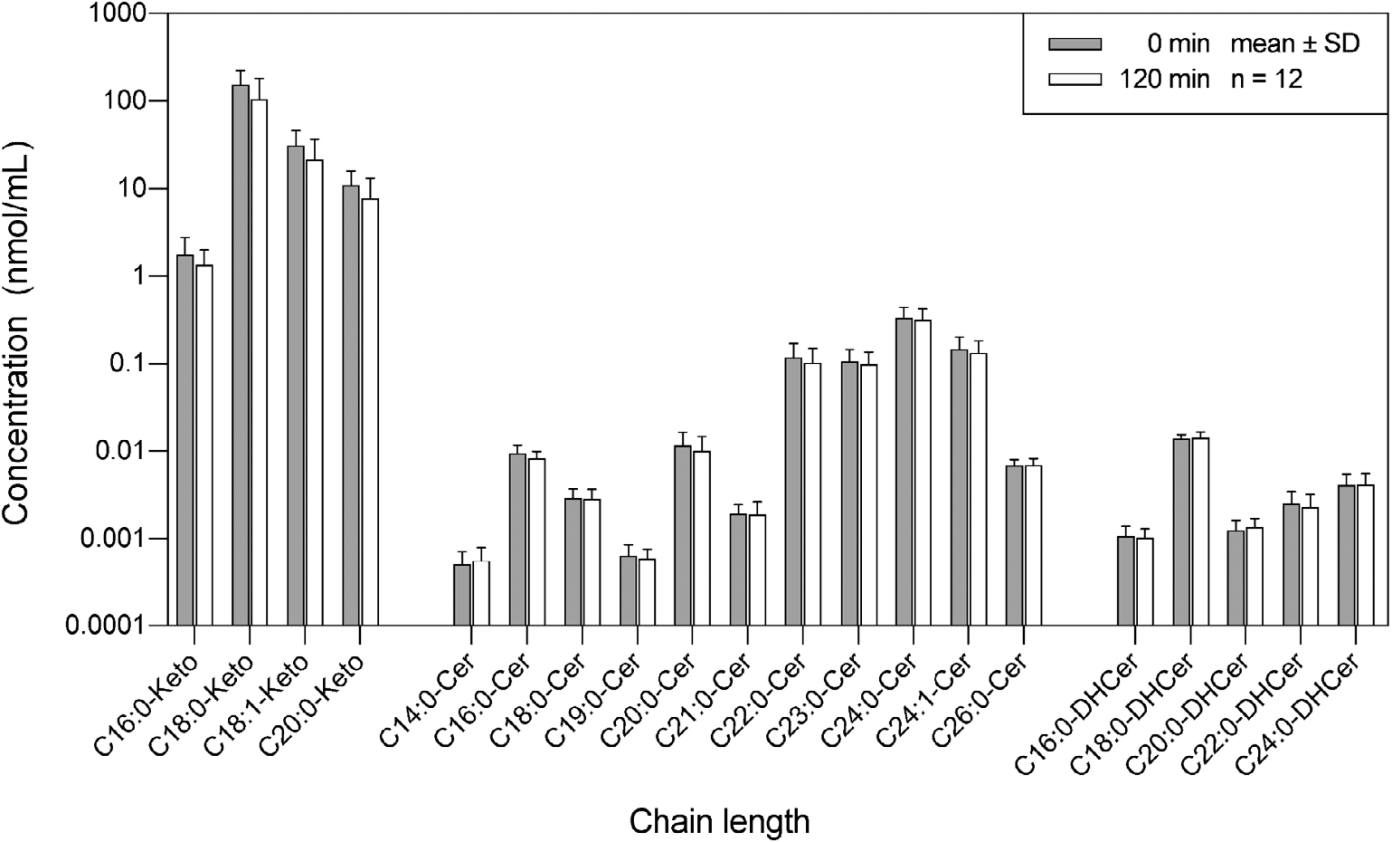
The distribution of sphingolipids in the de novo synthesis pathway in horses (n = 12), before (0 minute) and after (120 minutes) an oral glucose test. The concentration of each sphingolipid was presented as mean ± SD on a logarithmic scale. Cer, ceramide; DHCer, dihydroceramide; Keto, 3‐ketosphinganine

In the de novo synthesis pathway (Figure 2), none of the Keto, ceramides, and DHCer were significantly different before and after OGT. The C18:0‐ketosphinganine was the dominant species among all Keto and within the de novo synthesis pathway (0 minute: 153 ± 68.8 nmol/mL; 120 minutes: 105 ± 76.2 nmol/mL). The C24:0‐Cer was the dominant species among all ceramides (0 minute: 0.337 ± 0.108 nmol/mL; 120 minutes: 0.319 ± 0.103 nmol/mL). The C18:0‐DHCer was the dominant species among all DHCer (0 minute: 0.0139 ± 0.0016 nmol/mL; 120 minutes: 0.0143 ± 0.0025 nmol/mL).

**FIGURE 3:**
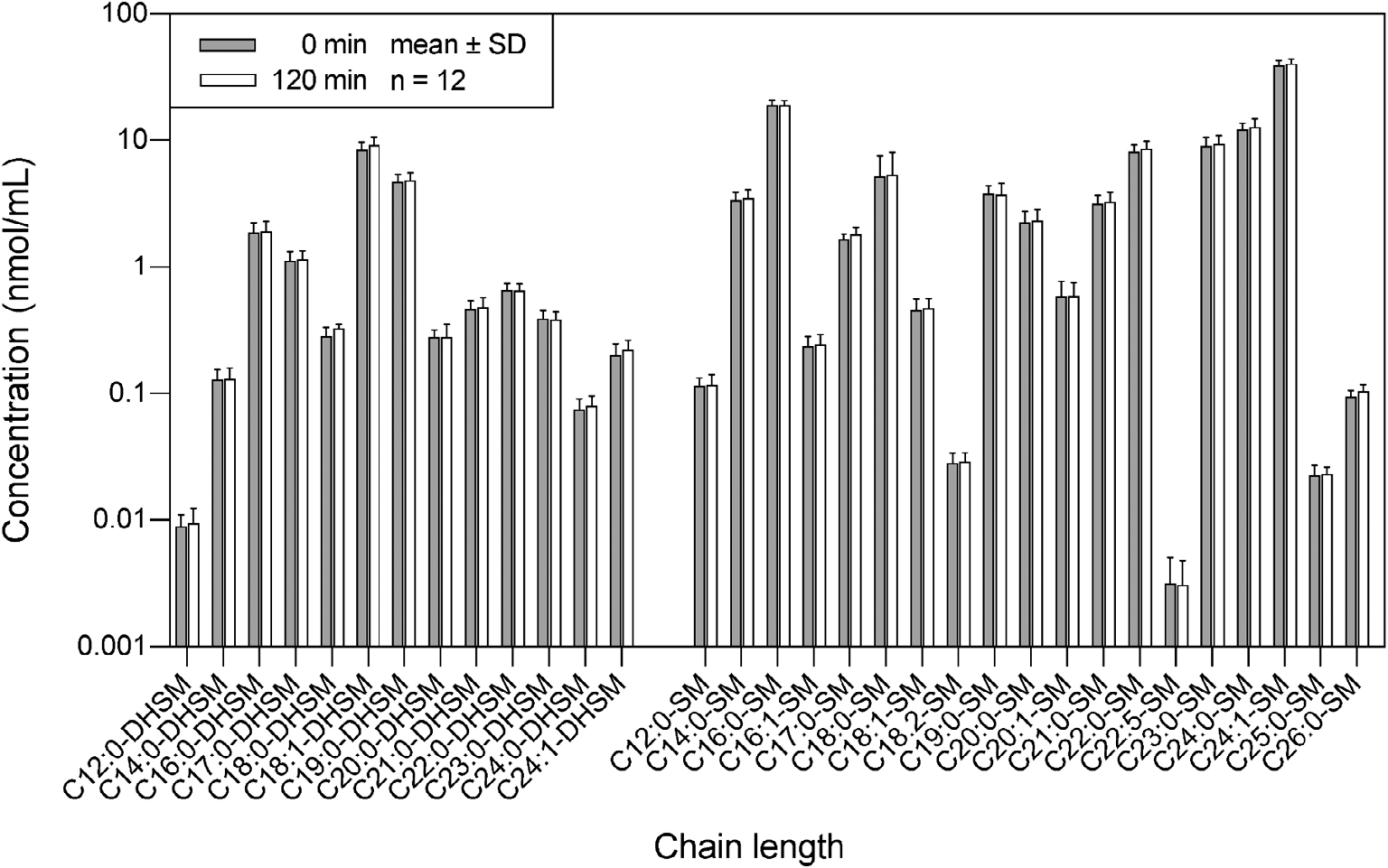
The distribution of sphingolipids in the sphingomyelinase pathway in horses (n = 12), before (0 minute) and after (120 minutes) an oral glucose test. The concentration of each sphingolipid was presented as mean ± SD on a logarithmic scale. DHSM, dihydrosphingomyelin; SM, sphingomyelin

In the SM pathway (Figure 3), none of the DHSM and SM were significantly different before and after OGT. The C18:1‐DHSM was the dominant species among the DHSM (0 minute: 8.36 ± 1.35 nmol/mL; 120 minutes: 9.15 ± 1.45 nmol/mL), and C24:1‐SM was the dominant species among the SM and within the SM pathway (0 minute: 12.2 ± 1.49 nmol/mL; 120 minutes; 12.8 ± 1.91 nmol/mL).

**FIGURE 4:**
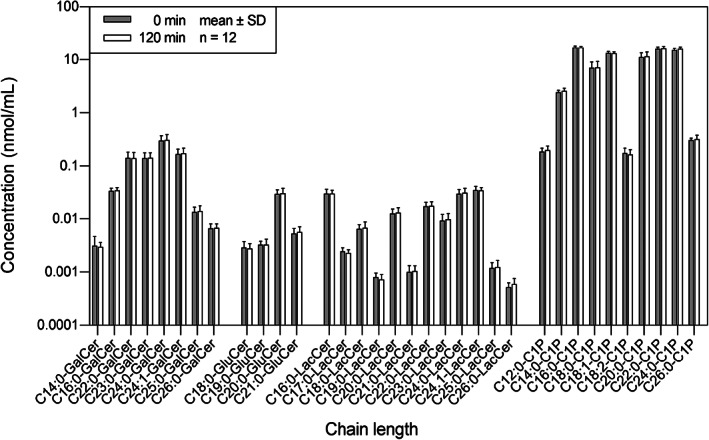
The distribution of sphingolipids in the salvage pathway in horses (n = 12), before (0 minute) and after (120 minutes) an oral glucose test. The concentration of each sphingolipid was presented as mean ± SD on a logarithmic scale. C1P, ceramide‐1‐phosphate; GalCer, galatosylceramide; GluCer, glucosylceramide; LacCer, lactosylceramide

In the salvage pathway (Figure 4), none of the GalCer, GluCer, LacCer, and C1P were significantly different before and after OGT. The C24:0‐GalCer was the dominant species among the GalCer (0 minute: 0.303 ± 0.0718 nmol/mL; 120 minutes: 0.311 ± 0.0780 nmol/mL). The C20:0‐GluCer was the dominant species among the GluCer (0 minute: 0.0298 ± 0.0058 nmol/mL; 120 minutes: 0.0309 ± 0.0072 nmol/mL). The C24:1‐LacCer was the dominant species among the LacCer (0 minute: 0.0351 ± 0.0062 nmol/mL; 120 minutes: 0.0349 ± 0.0046 mmol/mL). The C16:0‐C1P was the dominant species among the C1P and within the salvage pathway (0 minute: 17.1 ± 1.04 nmol/mL; 120 minutes: 17.0 ± 0.963 mmol/mL). Collectively, the sphingolipid profiles before and after OGT were not statistically different in the de novo synthesis pathway, SM pathway, and salvage pathway.

### 3.2 Insulin response during OGT

**FIGURE 5:**
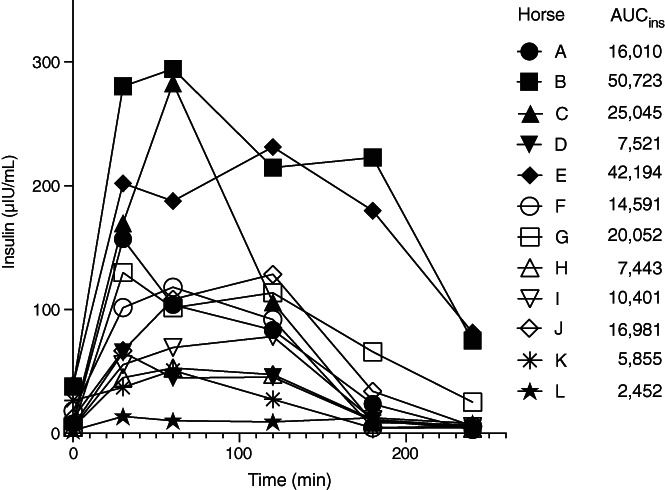
Individual insulin responses of horses during the oral glucose test (n = 12). Eight of 12 horses reached their insulin peak at 30 or 60 minutes after glucose administration. Ten of 12 horses returned their insulin concentration back to the baseline level at 240 minutes. AUC, area under the curve

As shown in Figure 5, plasma insulin concentrations increased rapidly from 0 to 30 minutes after glucose administration. Eight of 12 horses reached their insulin peak at 30 or 60 minutes. The insulin concentrations of 10 of 12 horses returned to baseline at 240 minutes, except for horses B and E. Collectively, horse B had the largest insulin response (AUC_ins_ = 50 723 μIU/mL × min), followed by horse E (AUC_ins_ = 42 194 μIU/mL × min) and horse C (AUC_ins_ = 25 045 μIU/mL × min).

### 3.3 Correlation of sphingolipid concentrations with AUC_ins_

**FIGURE 6:**
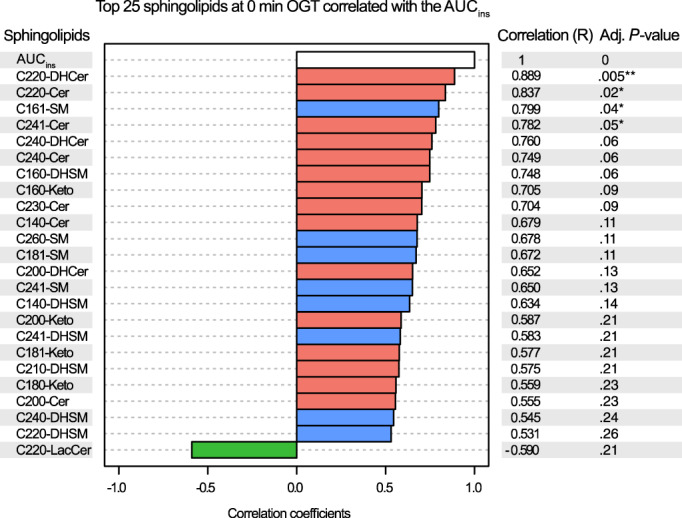
Top 25 sphingolipids measured at 0 minute of the oral glucose test (OGT), correlated with the AUC_ins_. Sphingolipids from the de novo synthesis pathway are in red, sphingomyelinase pathway in blue, and salvage pathway in green. *P*‐values were adjusted with FDR correction in Benjamini‐Hochberg procedure. Four species of sphingolipids were significantly positively correlated with AUC_ins_: C22:0‐DHCer (**P < .01), C22:0‐Cer, C16:1‐SM, and C24:1‐Cer (*P < .05). AUC, area under the curve; Cer, ceramide; DHCer, dihydroceramide; SM, sphingomyelin

To evaluate correlations between sphingolipids and the insulin response, 0 and 120 minutes concentrations of all sphingolipids were analyzed separately. The 25 sphingolipids most highly correlated with AUC_ins_, based on 0 minute sphingolipid concentrations, are presented in Figure 6. The C22:0‐DHCer showed the strongest correlation with insulin response, with the highest correlation coefficient (*R* = 0.889) and smallest adjusted *P*‐value (*P* = .005), followed by C22:0‐Cer (*R* = 0.837; *P* = .02), C16:1‐SM (*R* = 0.799; *P* = .04), and C24:1‐Cer (*R* = 0.782; *P* = .05). The C22:0‐LacCer was the only sphingolipid among these that was negatively correlated with AUC_ins_ (*R* = −0.590; *P* = .21).

**FIGURE 7:**
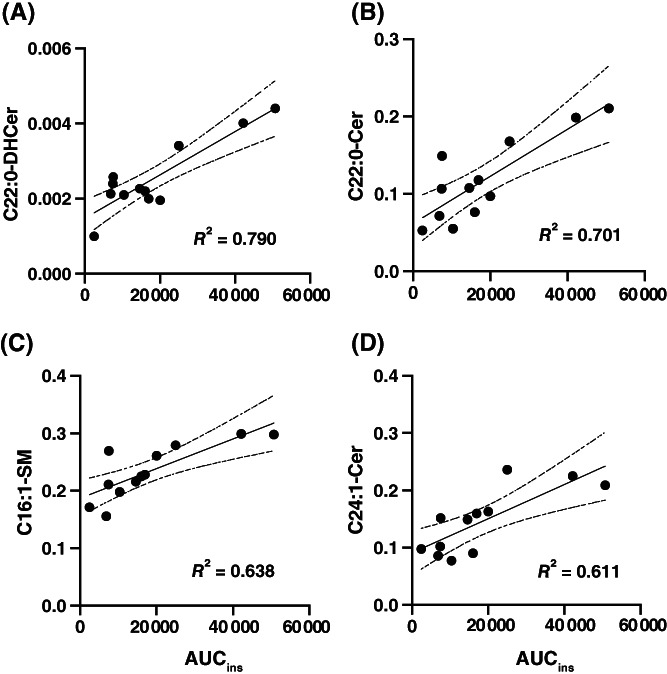
The simple linear regression of sphingolipids measured at 0 minute, with AUC_ins_ during the oral glucose test. A, C22:0‐DHCer; B, C22:0‐Cer; C, C16:1‐SM; D, C24:1‐Cer. AUC, area under the curve; Cer, ceramide; DHCer, dihydroceramide; SM, sphingomyelin

The simple linear regression plots of the sphingolipids with *P* < .05 are shown in Figure 7. The C22:0‐DHCer had the most significant line of best fit (Figure 7A; *R* ^2^ = 0.790), followed by C22:0‐Cer (Figure 7B; *R* ^2^ = 0.701), C16:1‐SM (Figure 7C; *R* ^2^ = 0.638), and C24:1‐Cer (Figure 7D; *R* ^2^ = 0.611).

**FIGURE 8:**
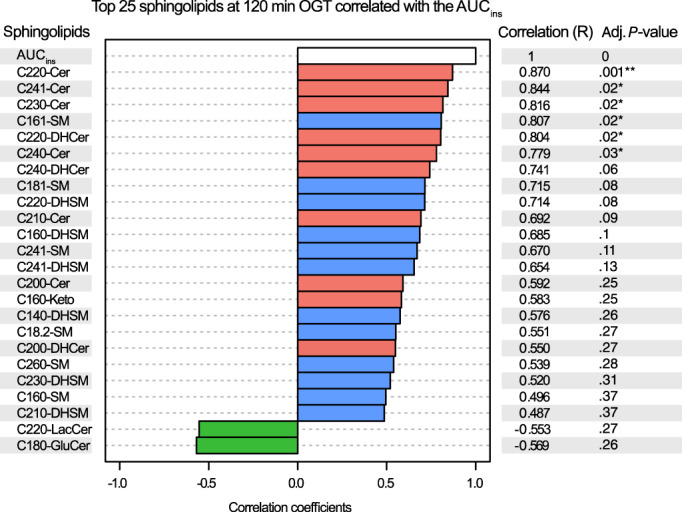
Top 25 sphingolipids measured at 120 minutes of the oral glucose test (OGT), correlated with the AUC_ins_. Sphingolipids from the de novo synthesis pathway are in red, sphingomyelinase pathway in blue, and salvage pathway in green. *P*‐values were adjusted with FDR correction in Benjamini‐Hochberg procedure. Six species of sphingolipids were significantly positively correlated with AUC_ins_: C22:0‐Cer (**P < .01), C24:1‐Cer, C23:0‐Cer, C16:1‐SM, C22:0‐DHCer, and C24:0‐Cer (*P < .05). AUC, area under the curve; Cer, ceramide; DHCer, dihydroceramide; SM, sphingomyelin

Figure 8 shows the 25 sphingolipids most highly correlated with AUC_ins_, based on the 120 minutes concentration of sphingolipids. The C22:0‐Cer showed the highest correlation with insulin response, with the highest correlation coefficient (*R* = 0.870) and the lowest adjusted *P*‐value (*P* = .001), followed by C24:1‐Cer (*R* = 0.844; *P* = .02), C23:0‐Cer (*R* = 0.816; *P* = .02), C16:1‐SM (*R* = 0.807; *P* = .02), C22:0‐DHCer (*R* = 0.804; *P* = .02), and C24:0‐Cer (*R* = 0.779; *P* = .03). Two of these 25 sphingolipids were negatively correlated with AUC_ins_: C22:0‐LacCer (*R* = −0.553; *P* = .27) and C18:0‐GluCer (*R* = −0.569; *P* = .26).

**FIGURE 9:**
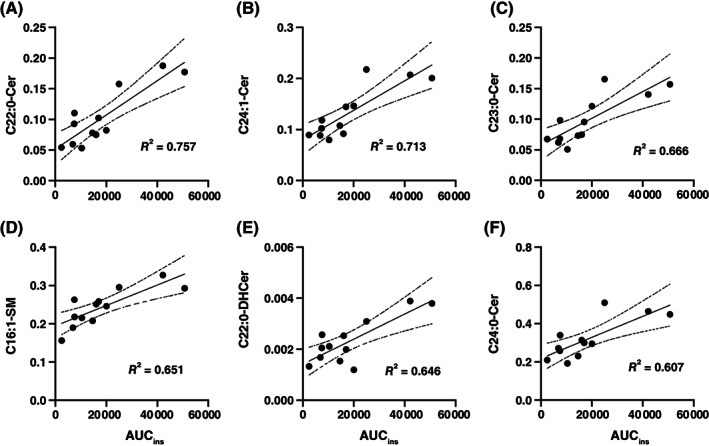
The simple linear regression of sphingolipids measured at 120 minutes, with AUC_ins_ during the oral glucose test. A, C22:0‐Cer; B, C24:1‐Cer; C, C23:0‐Cer; D, C16:1‐SM; E, C22:0‐DHCer; F, C24:0‐Cer. AUC, area under the curve; Cer, ceramide; DHCer, dihydroceramide; SM, sphingomyelin

The simple linear regression plots of the sphingolipids with *P* < .05 are shown in Figure 9. The C22:0‐Cer had the most significant line of best fit (Figure 8A; *R* ^2^ = 0.757), followed by C24:1‐Cer (Figure 8B; *R* ^2^ = 0.713), C23:0‐Cer (Figure 8C; *R* ^2^ = 0.666), C16:1‐SM (Figure 8D; *R* ^2^ = 0.651), C22:0‐DHCer (Figure 8E; *R* ^2^ = 0.757), and C24:0‐Cer (Figure 8F; *R* ^2^ = 0.607).

## 4 DISCUSSION

Our objectives were to investigate the impact of OGT on sphingolipid metabolism and to identify sphingolipids that are correlated with the insulin response in horses. Our findings showed that the OGT did not acutely alter the sphingolipid profile over a period of 120 minutes. However, a higher insulin response was associated with higher SM, ceramide, and DHCer concentrations, implying that insulin‐dysregulated horses likely had upregulated sphingolipid metabolism. Ours is the first study to provide absolute plasma concentrations of the comprehensive sphingolipid metabolome, covering the various sphingolipid pathways, in horses with a characterized insulin response during OGT. Building on previous metabolomics studies showing that a higher insulin response in horses was significantly associated with a higher plasma glycerophospholipid concentration and lower plasma arginine, acylcarnitines, spermidine, trans‐4‐hydroxyproline and methionine sulfoxide concentration,^19^, ^27^ our findings further characterize the metabolic phenotype of insulin‐dysregulated horses.

### 4.1 Limited acute impact of the OGT on sphingolipid metabolism

We observed that the sphingolipid metabolism was not significantly changed after glucose administration by comparing the sphingolipid profiles between 0 and 120 minutes. The acute challenge of OGT may not have a clinically relevant impact on sphingolipid metabolism, a finding that is in agreement with the results of a previous metabolomics study conducted using a PO sugar test in Welsh Ponies^28^ that found that palmitoyl SM concentration did not differ statistically between 0 and 75 minutes of the PO sugar test. Furthermore, another study^19^ showed that none of the 15 investigated species of sphingolipid were significantly changed after an acute OGT in horses. These studies collectively confirm the limited impact of an acute sugar load on sphingolipid metabolism in horses. Our findings further extend our understanding that, in addition to SM, other sphingolipid metabolites such as ceramides, glycosylceramides, and phosphorylated sphingolipids also remain unaffected by an acute OGT.

It is possible however that long‐term nutritional interventions, such as high carbohydrate diets or repeated sugar administration, could disrupt sphingolipid metabolism.^29^, ^30^ In cats, a plasma metabolomics study showed that plasma C18:1‐, C24:0‐Cer, and C18:1‐, C24:0‐SM concentrations were significantly decreased in the glucose supplementary diet group after a 21‐day feeding regimen.^31^ The concentrations of ceramides and SM also were decreased in dogs on a glucose supplementary diet in the same study. In rats, plasma sphinganine‐1‐phosphate and sphingosine‐1‐phosphate concentrations, but not ceramide concentrations, were significantly higher in the high‐carbohydrate diet rats than in the control rats over a period of 8 weeks of feeding,^30^ whereas the concentrations of pancreatic ceramides and sphingosines were significantly higher in the treatment group than in the controls. These studies collectively showed the long‐term impact of dietary glucose or carbohydrates on sphingolipid metabolic pathways in various species. Ceramides, SM, and phosphorylated sphingolipids are the sphingolipid species that were significantly affected by dietary interventions. Interestingly, ceramides (C22:0‐, C23:0‐, C24:0‐, C24:1‐Cer) and SM (C16:1‐SM) also were significantly correlated with the insulin response in horses in our study. Although our results showed that an acute challenge of OGT did not alter the sphingolipid profiles after 120 minutes, the impact of glucose load on sphingolipid metabolism should be further evaluated in horses using a longer duration of dietary challenge. Considering that sphingolipid metabolic pathways are evolutionarily conserved in eukaryotes,^32^ it is anticipated that plasma sphingolipid profiles in horses would have higher phosphorylated sphingolipid concentrations, and slightly lower ceramide and SM concentrations because of a longer term dietary carbohydrate load.

### 4.2 The pathophysiological role of ceramides, SM, and DHCer in horses

We observed that ceramides, SM, and DHCer were positively correlated with the insulin response AUC_ins_. Interestingly, these sphingolipid species were biochemically connected in the SM pathway and the last step of the de novo synthesis pathway (Figure 1), suggesting upregulation of sphingolipid metabolism in horses with impaired insulin regulation. Ceramides are essential lipids in eukaryotes that are involved in signal transduction of apoptosis,^33^ the inflammatory response,^2^ and insulin signaling.^34^ With regard to the insulin signaling pathway, ceramides attenuate insulin action by stimulating the dephosphorylation of Akt, by releasing the inhibitory function of protein phosphatase 2A and by blocking Akt trafficking through protein kinase Cζ inactivation.^35^ In our study, the positive correlation of insulin response and ceramide concentration likely was a consequence of the direct molecular actions of ceramides on components of the insulin signaling pathway. Based on the regulatory role of ceramides, observation of high plasma ceramide concentrations in horses could indicate a higher risk of ID. This strong association between plasma ceramide concentration and ID also was reported in insulin‐dysregulated mice and diabetic humans,^36^, ^37^ suggesting that plasma ceramide concentration could be developed as a biomarker of insulin sensitivity in mammals, subject to further validation. Additional experiments in horses focusing on the assessment of tissue insulin sensitivity and resistance combined with ceramide analyses are required to prove this hypothesis. We also observed that the very‐long‐chain ceramides (C22:0‐, C23:0‐, C24:0‐, C24:1‐Cer) had the highest correlation coefficient, compared with other chain lengths of ceramides, indicating higher ceramide synthase 2 (CerS2) expression in insulin‐dysregulated horses. This observation aligns with a previous study that reported CerS2 as the dominant CerS in cattle, suggesting that CerS2 could be a major enzyme in modulating insulin sensitivity.^38^

Chemically, SM are a modification of ceramides, having an additional phosphate‐containing polar group. Physiologically, SM are recognized as a source of bioactive molecules.^39^ In response to the specific type of agonist or cytokine, such as tumor necrosis factor alpha, SM, an enzyme that converts SM to ceramides, is activated and increases the abundance of ceramides for the corresponding physiological responses.^40^ Although a previous study identified the regulatory role of SM on apoptotic signaling pathways,^41^ SM themselves may not have a primary regulatory role in the insulin signaling pathway. Another study found that the insulin regulatory effect in the SM pathway was accomplished by ceramides being converted from SM.^42^ Thus, higher plasma SM concentrations in horses may not precisely reflect metabolic health status because the pathophysiological role of SM in developing ID is less clear than that of ceramides.^42^ However, in combination with the increase in ceramide concentrations, a decrease in plasma concentration of SM could be an indicator of an upregulated SM pathway and perturbed sphingolipid metabolism, suggesting a higher risk of ID.

Dihydroceramides are the precursors of ceramides in the final step of the de novo synthesis pathway (Figure 1). This step converts DHCer into ceramides by adding a 4,5‐trans double bond on the sphingoid backbone. This double bond structurally differentiates ceramides from DHCer, and affects the cell signaling pathway. A previous study determined that C2‐Cer, but not C2‐DHCer, induced apoptosis in human monoblastic leukemia cells.^43^ Another study showed that the absence of the double bond could restore insulin sensitivity and produce hepatic steatosis in DHCer desaturase 1 knock‐out mice, suggesting an essential role of the double bond on apoptosis and insulin signaling.^44^ Dihydroceramides formerly were thought to be biologically inactive, at least relative to ceramides. However, more recent findings suggest that DHCer might be involved in cell signaling events such as autophagy and proliferation,^45^ even if DHCer are not likely to modulate insulin response. Thus, an observation of high plasma DHCer concentration in our study may not be closely linked to metabolic health status, because the DHCer do not appear to play a regulatory role in the insulin signaling pathway.^44^ However, in conjunction with an increase in ceramide concentrations, a high concentration of DHCer could indicate upregulated sphingolipid metabolism and suggest metabolic imbalance.

A potential limitation of our study is that our observations were limited to Icelandic horses. Therefore, we cannot conclude that our findings can be generalized to other breeds, although the link between insulin resistance and ceramide synthesis seems to be evolutionarily conserved in mammals.^3^ Because our study population was small (n = 12), larger scale studies using more replicates, and preferably using various breeds, are necessary to extrapolate our data to larger populations of horses. Additional mechanistic experiments focusing on the link between cellular sphingolipid metabolism and insulin resistance are warranted to confirm causative relationships.

## 5 CONCLUSION

Our study is the first to present comprehensive plasma sphingolipid profiles in horses in the context of insulin response during OGT. Our findings help elucidate the relationship between sphingolipid metabolism and insulin response in horses. Six species of sphingolipids were evaluated, including ceramides, SM, and DHCer, providing potential for further investigations in biomarker development and inclusion into diagnostic panels for assessment of ID in horses. However, the pathophysiological role of the identified sphingolipids on developing ID in horses is not yet fully explained. In vitro studies using various sphingolipid species are warranted to elucidate the complex mechanistic regulatory relationships between sphingolipid metabolism and insulin response in horses.

## CONFLICT OF INTEREST DECLARATION

Authors declare no conflict of interest.

## OFF‐LABEL ANTIMICROBIAL DECLARATION

Authors declare no off‐label antimicrobial use of antimicrobials.

## INSTITUTIONAL ANIMAL CARE AND USE COMMITTEE (IACUC) OR OTHER APPROVAL DECLARATION

The State Office for Consumer Protection and Food Safety (LAVES) approved the study in accordance with the German Animal Welfare Law (Ref: 33.19‐42502‐04‐18/3006).

## HUMAN ETHICS APPROVAL DECLARATION

Authors declare human ethics approval was not needed for this study.
